# Reactive Paper Spray Mass Spectrometry Enables Speciation of Trace Levels of Mercuric Halides

**DOI:** 10.1021/acs.analchem.6c02808

**Published:** 2026-06-01

**Authors:** Mohammad Borna Bahramsari, Md Tanim-Al Hassan, Hao Chen, Alexei F. Khalizov

**Affiliations:** † Department of Chemistry and Environmental Science, 5965 New Jersey Institute of Technology, 161 Warren Street, Newark, New Jersey 07102, United States; ‡ Department of Chemical and Materials Engineering, New Jersey Institute of Technology, 161 Warren Street, Newark, New Jersey 07102, United States

## Abstract

Gaseous oxidized mercury (GOM) plays a key role in atmospheric mercury cycling, yet its specific molecular forms remain known mostly from theoretical calculations. Existing analytical methods relying on preconcentration and thermal desorption erase the original chemical speciation of this ultratrace pollutant, providing only indirect evidence. Here, we report a nonthermal, reagent-directed ionization strategy based on reactive paper spray mass spectrometry (RPS-MS), allowing us to recover the chemical identity of preconcentrated mercuric halides. The ionization is initiated by the addition of a halide reagent ion and proceeds through reversible halide addition and loss steps that drive ligand-exchange reactions characteristic of mercuric halides in solution and on surfaces. The mechanism is elucidated through kinetic modeling and experimentally validated by observing ionic products of halide addition and halide loss. By comparing iodide and chloride as ionizing reagents, we show that a higher degree of ionization can be achieved with iodide, using a significantly lower concentration and at the expense of only moderate overexchange. This reagent-controlled chemistry offers a generalizable framework that can be extended to atmospheric GOMs and other ultratrace oxidized metal systems, as demonstrated by speciating an ambient air sample and a CdCl_2_ standard.

## Graphical abstract

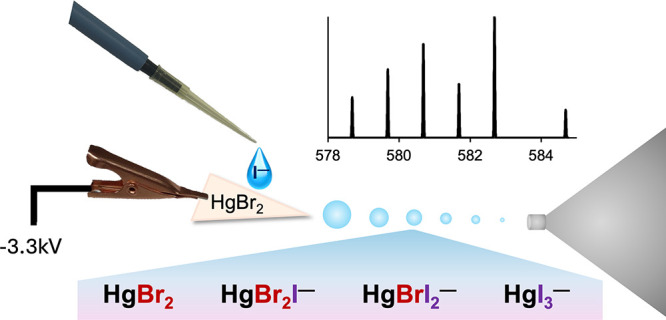

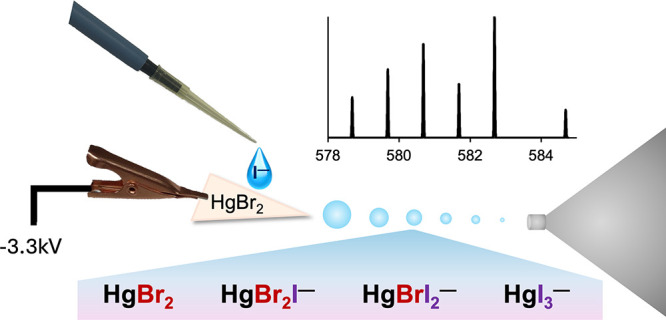

## Introduction

Gaseous oxidized mercury (GOM) represents a family of mercuric chemicals that play crucial roles in cycling of atmospheric mercury, yet elucidating molecular identities of GOM remains a significant analytical challenge because of its ultratrace atmospheric abundance and chemical lability. As a result, current understanding of GOM speciation and reactivity relies largely on quantum chemical predictions rather than direct experimental observation, limiting mechanistic insights into processes governing mercury oxidation, interconversion, and transport in the environment.^1–3^

According to our current understanding, atmospheric GOM is formed in multistep oxidation pathways of gaseous elemental mercury (Hg(0)) emitted by fossil fuel combustion, incineration, cement production, and artisanal mining.^4,5^ The initial step involves pressure-dependent termolecular association reactions between Hg(0) and atmospheric radicals, most notably bromine (Br^•^) and, to a lesser extent, hydroxyl (OH^•^), forming thermally and photolytically labile mercurous radical intermediates such as HgBr^•^ and HgOH^•^.^6–10^ These Hg­(I) species undergo further oxidation by ozone (O_3_) or relatively abundant radicals, including NO_2_ and HO_2_, yielding more stable divalent mercury compounds commonly proposed as constituents of the operational GOM pool, including XHgONO, XHgOOH, XHgONO_2_, and XHgOH, where X represents either Br or OH.^11,12^ Knowledge of the chemical speciation is critical because it governs key physicochemical properties of GOM, including photolytic reactivity, thermal and chemical stability in gas and particle phases, and volatility. These properties can vary substantially among different GOM species, leading to markedly different atmospheric lifetimes and fates. For example, HgCl_2_ produced through the conversion of the original GOM on atmospheric aerosols^13^ is nonphotolabile under tropospheric conditions, whereas BrHgNO_2_ can undergo efficient photolysis, resulting in fundamentally different atmospheric processing pathways.^7,14^ Recent modeling studies indicate that nearly half of atmospheric GOM could be HgCl_2_ formed through aerosol processing.^7^

The ultratrace atmospheric concentrations of GOM (pg/m^3^) makes its detection a significant analytical challenge.^1–3^ Although very recently the in situ online detection of individual mercuric halides in the polar boundary layer has been reported by atmospheric pressure chemical ionization mass spectrometry (APCI-MS),^15^ the observed speciation deviates from current model predictions. Furthermore, implementing this direct method in polluted urban environments may be far from straightforward, necessitating preconcentration on sorbents followed by thermal desorption, which is the de facto method that enables GOM analysis.^1^ A sorbent must quantitatively capture GOM and quantitatively release it under mild conditions—two inherently conflicting requirements in one material. Among the known sorbents are nylon membranes, metal oxide glasses, and custom-made traps packed with various materials, such as shredded PFA, poly­(dimethylsiloxane), quartz wool, and nanoparticles derivatized with polysulfides.^16–18^ Even after preconcentration, the sample contains only a few nanograms of GOM spread across multiple possible species, making mass spectrometry (MS), with its high sensitivity and molecular specificity, a logical choice for analyzing the released compounds. Gas chromatography with electron impact MS and atmospheric pressure ionization MS have been evaluated,^17,18^ but reliable detection was achieved only for HgCl_2_ and HgBr_2_. It is likely that for species other than mercuric halides, surface reactions and thermal decomposition during sample heating obscure the identity of collected GOM. Consequently, the most successful current approach bypasses mass spectrometry entirely: it converts the thermally released chemicals into elemental mercury, which is then detected by atomic fluorescence spectroscopy.^19^ Chemical speciation is inferred with this method, albeit indirectly, by deconvoluting the evolving GOM sample thermogram using a set of thermograms obtained for several individual Hg­(II) standards.^19,20^ It is unrealistic to expect that ambient GOM can be represented well by a set of commercially available mercury standards, but an ability to speciate GOM even at this level of detail is a significant analytical achievement. Although this approach has been widely employed to study GOM speciation worldwide,^21,22^ there is a clear need for a nondestructive method of releasing GOM species from preconcentrated samples in readily detectable forms.

Paper spray mass spectrometry (PS-MS) was introduced in 2010 by the group of Cooks^23^ as an ambient ionization technique that enables rapid, direct analysis of samples deposited on cellulose filters and similar media without extensive preparation, thereby minimizing analyte alteration during analysis. This approach has found broad application in trace-level analysis in the context of biological, environmental, and forensic sciences as well as in synthetic chemistry:^24–28^ a complex matrix containing a chemical of interest, such as a droplet of blood or urine laced with cocaine can be placed on a small triangular piece of treated hydrophobic paper filter and interrogated without any additional pretreatment.^29^ We hypothesize that PS-MS can serve as an alternative, nonthermal route for probing the composition of GOM collected on sorbents under minimally perturbative conditions. Here, “nonthermal” refers to the absence of bulk thermal desorption of analytes from the collection substrate, distinguishing PS-MS from conventional sorbent-based thermal desorption approaches.

Unlike many other analytes readily ionizable by simple pH adjustment,^25,30^ mercuric chemicals do not dissociate in common organic solvents or even in water, or their dissociation is accompanied by rapid hydrolysis.^31^ Reactive paper spray mass spectrometry (RPS-MS) has been used with such poorly ionizable species by performing *in situ* chemical transformation to convert them into readily charged derivatives, e.g., reacting quinones and formaldehyde with nucleophilic reagents cysteamine and hydrazides helped facilitating efficient ion formation during paper spray ionization.^32,33^ In the context of mercuric chemicals Hg­(II), a complexation reaction with an appropriate ligand, L^–^, can be employed to produce an ionic form while preserving speciation. As soft Lewis acids, Hg­(II) species readily react with soft Lewis bases, such as halides to form charged complexes Hg­(II)­L^–^.^2^ Stability of these complexes depends strongly on the nature of the halide, and in contrast to the gas phase, where Hg­(II)–F^–^ bond is the strongest,^34,35^ in solution the order is reversed and Hg­(II) forms complexes more favorably with iodide (I^–^) than with fluoride (F^–^).^36,37^ Hence, conducting experiments with different halide reagent ions can enable species-resolved detection and quantitative analysis of GOM, while providing mechanistic insights into halide-dependent complexation pathways. Recent work from our group has shown that nitrite-based mercuric species exhibit low volatility, suggesting that RPS-MS may be the only approach for their detection once collected on sorbents.^38^

Here, we demonstrate a reagent-directed PS-MS approach that enables direct, species-resolved detection, overcoming limitations inherent to existing methods for GOM speciation that are based on thermal desorption. As a proof of concept, we rely on HgCl_2_, HgBr_2_, and HgI_2_ as GOM surrogates and use iodide and chloride ions as ionizing reagents (dopants). By directly observing mixed-halide ionic intermediates formed through reversible ligand-exchange processes and supporting these observations with kinetic modeling, we establish a unified mechanistic framework for the halide-driven complexation of mercuric species under ambient ionization conditions. As a demonstration of the new method, we successfully speciated halide-containing Hg­(II) species and quantified Hg­(II) in a sample of reactive mercury (RM) collected from ambient air, where RM is a sum of GOM and particulate-bound mercury (PBM). Additionally, we speciated a CdCl_2_ standard to show that the method can be extended to other metals.

## Methods

### Chemicals and Materials

Mercuric bromide (HgBr_2_, Alfa Aesar, >99%), mercuric chloride (HgCl_2_, Honeywell, >99%), and mercuric iodide (HgI_2_, Sigma-Aldrich, ACS reagent >99%) were used as GOM surrogates. Cadmium chloride (CdCl_2_, FisherChemical, >99.7%) was used to demonstrate the applicability of the method to other metals. Ammonium iodide (NH_4_I, Thermo Scientific, 99%) and ammonium chloride (NH_4_Cl, Thermo Scientific, 99%) were used as ionization reagents (dopants). Methanol (CH_3_OH, Sigma-Aldrich, ACS-grade LC reagent, ≥99.9%) was used as the solvent. Chromatography paper (Whatman 4CHR) was purchased from Cytiva. Perfluorooctanoic acid (PFOA, CF_3_(CF_2_)_6_COOH, Sigma-Aldrich, 95%) and perfluorononanoic acid (PFNA, CF_3_(CF_2_)_7_COOH, Sigma-Aldrich, 97%) were used as internal standards during calibration.

### RPS-MS Analysis

Paper substrates were cut into isosceles triangles (10 mm base and 10 mm height) using a CO_2_ laser, then washed and sonicated in a 1:1 methanol–water solution. The substrates were subsequently dried in an oven at 70 °C prior to use. Before each experiment, 5–10 μL of the mercuric halide solution was deposited onto the paper and allowed to dry completely. Larger volumes were deposited in multiple additions with complete drying between each addition. The internal standard (PFOA for the low-resolution system and PFNA for the high-resolution system) was then deposited and dried in the same manner. During analysis, 20 μL of the reagent solution was applied to the paper for each elution. A spray voltage of −3.3 kV was used, with the tip of the paper, held by a flat-jaw copper alligator, positioned approximately 20–25 mm from the MS inlet to suppress corona discharge and minimize the signal background (Figure 1a). The sheath gas was turned off, and the heated ion transfer capillary was maintained at 170 °C. The ion transit time through the capillary is very short, minimizing the likelihood of thermal dissociation during transfer. Based on an estimate using typical complex binding energies (>20 kcal/mol) and a transit time of approximately 0.2 ms, we estimate that more than 99.9% of the ions survive capillary transfer. Consistent with this, we did not observe a significant decrease in the ion signal when the capillary temperature was increased from 170 to 270 °C.

**Figure 1:**
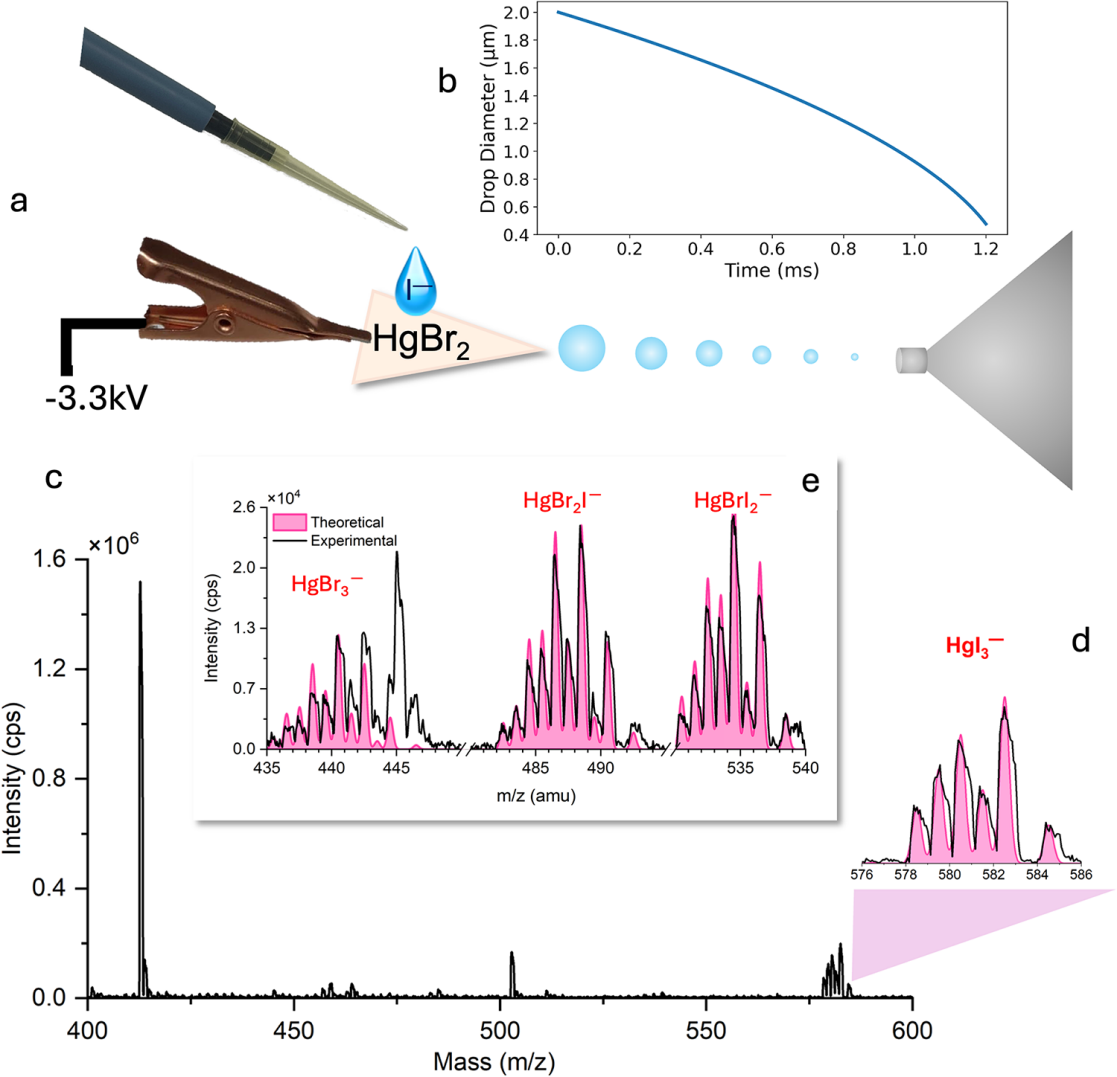
Detection of mercuric bromide by reactive paper spray mass spectrometry. (a) Experimental configuration illustrating deposition of the HgBr_2_ solution onto a paper substrate followed by negative-ion paper spray operated at −3.3 kV, generating charged microdroplets that undergo evaporation and form ions prior to mass spectrometric sampling. (b) Modeled time evolution of the spray droplet diameter during travel from the paper tip to the ion sampling cone, demonstrating rapid solvent evaporation on the millisecond time scale. (c) Low-resolution mass spectra of 14.4 ng HgBr_2_ during the first elution, obtained with two different iodide concentrations. In the presence of 31.5 μM NH_4_I, only HgI_3_^–^ is detected and no other mercury-containing ions are observed; the magnified HgI_3_^–^ signal is also shown in the inset (d). (e) At a reduced iodide concentration of 3.15 μM NH_4_I, partial halide-exchange ion products HgBr_3_^–^, HgBr_2_I^–^, and HgBrI_2_^–^ can be observed. To isolate mass spectra of these ion products, background contributions were removed by subtracting the mass spectrum acquired during a subsequent elution using excess iodide, which ensured complete conversion of all mercuric forms to HgI_3_^–^.

Low-resolution measurements were performed on a TSQ Quantum Classic triple-quadrupole mass spectrometer (Thermo Scientific). High-resolution measurements were performed on a Q Exactive Orbitrap mass spectrometer (Thermo Scientific). The triple-quadrupole instrument operated at a peak width of 0.7 amu, whereas the Orbitrap resolving power of 140,000 at *m*/*z* 200 corresponds to a peak width of approximately 0.0014 amu.

Speciation experiments were conducted using iodide and chloride reagent ions by low- and high-resolution MS. In the low-resolution system, 14.4 ng of deposited mercuric bromide, corresponding to 4 μM initial concentration on paper during first elution, was speciated using either 3.15 μM iodide or 300 μM chloride. Under high-resolution MS, 3.6 ng of deposited mercuric bromide (1 μM) was speciated using either 0.16 μM iodide or 59.8 μM chloride. Similar loadings were used during the analysis of mercuric chloride and mercuric iodide.

Quantification was performed using a large excess of iodide over mercuric bromide to convert all mercury to HgI_3_^–^. Since paper spray is a complex ambient ionization technique, the absolute signal can vary with humidity, sample matrix, paper-to-cone distance, paper-to-paper differences, and other factors.^39^ To minimize this variability, a constant amount of internal standard was added to the paper substrate during each analysis, and the calibration curve was constructed from the ratio of the HgI_3_^–^ signal to that of the internal standard. The calibration was performed using both the low-resolution and high-resolution instruments, and there were two key differences between the calibration procedures for these two platforms. First, the mercury loading was lower in the high-resolution measurements, allowing a lower iodide concentration during elution (26.2 μM versus 31.5 μM used for the low-resolution detection). Second, the internal standard differed between platforms. PFOA, used during low-resolution calibration, exhibited substantial background in the high-resolution system. Hence, PFNA was adopted as the internal standard for the latter to ensure clean background and reproducible quantification. For low-resolution calibration, seven mercury loadings were used, ranging from 0.9 to 14.4 ng. For high-resolution calibration, five loadings were used, ranging from 0.18 to 3.6 ng. The internal standard loading was held constant for all calibration points: 16.7 ng PFOA for the low-resolution system and 12.5 ng PFNA for the high-resolution system. The limit of detection (LOD) was determined as 3σ/*S*, where σ is the standard deviation of the signal at the lowest loading level and *S* is the sensitivity obtained from the calibration curve. All calibration curve measurements were performed in triplicate.

### Kinetic Model

A kinetic model was developed to describe halide-mediated ligand-exchange of HgBr_2_ in evaporating methanol microdroplets during reactive paper spray ionization, using either iodide or chloride as the reagent ion. The mechanism included all reversible steps connecting HgBr_2_ to HgL_3_^–^ (L is I, Cl or Br) through sequential Br^–^/L^–^ substitution.

Forward bimolecular halide addition rate constants were estimated using the Smoluchowski diffusion-limited expression, as suggested in previous work.^40^

1 $$k_{+}=4\pi R_{c}\left(D_{1}+D_{2}\right)$$

where *R*_c_ = 5.9–6.8 Å is the encounter radius and *D*_1_ and *D*_2_ are the diffusion coefficients of the two reacting species. Diffusion coefficients were calculated using the Stokes–Einstein relation

2 $$D=\frac{k_{B}T}{6\pi \eta r}$$

where *k*_B_ is the Boltzmann constant, *T* = 298 *K* is the temperature, η = 5.37 × 10^–4^ Pa s is the dynamic viscosity of methanol, and *r* = 1.8–4.7 Å is the hydrodynamic radius of the ions or molecules. These expressions yielded similar forward rate constants for all species, near 1.6 × 10^10^ L mol^–1^ s^–1^ in methanol.

Reverse rate constants, *k*_–_ were calculated from these estimated diffusion-limited forward rate constants, *k*_+_, and experimentally measured stability constants *K* for the trihalide complexes (Table S1):^41^

3 $$k_{-}=\frac{k_{+}}{K}$$

R1 $${\mathrm{HgI}}_{2}+I^{-}⇌{\mathrm{HgI}}_{3}^{-},\left(K\left(I\right)=1.8\times 10^{5}\;L\;{\mathrm{mol}}^{-1}\right)$$

R2 $${\mathrm{HgBr}}_{2}+{\mathrm{Br}}^{-}⇌{\mathrm{HgBr}}_{3}^{-},\left(K\left(\mathrm{Br}\right)=1.2\times 10^{3}\;L\;{\mathrm{mol}}^{-1}\right)$$

R3 $${\mathrm{HgCl}}_{2}+{\mathrm{Cl}}^{-}⇌{\mathrm{HgCl}}_{3}^{-},\left(K\left(\mathrm{Cl}\right)=19\;L\;{\mathrm{mol}}^{-1}\right)$$

Because mixed-halide stability constants are not available, all reverse rate constants for ions such as HgBr_2_I^–^ and HgBrI_2_^–^ were assigned based on the identity of the departing halide (Br^–^, I^–^, or Cl^–^) using the corresponding *K* value.

The microdroplet was modeled as a shrinking sphere with an initial diameter of 2 μm. Its time-dependent diameter *D*_drop_(*t*) followed the classical diffusion-limited evaporation model

4 $$\frac{dD_{\mathrm{drop}}}{dt}=-\frac{4D_{g}M}{\rho \mathrm{RT}}\frac{p}{D_{\mathrm{drop}}}$$

where *D*_g_ = 1.66 × 10^–5^ m^2^ is the gas-phase diffusion coefficient of methanol vapor in air, *M* = 32 × 10^–3^ kg mol^–1^ is the molar mass of liquid methanol, ρ = 792 kg m^–3^ is the liquid density, *R* is the gas constant, and *T* = 298 K is the temperature. The parameter *p* is the net vapor-pressure driving force,

5 $$p=P_{\mathrm{sat}}\left(1-S\right)$$

where *P*_sat_ = 1.32 × 10^4^ Pa is the saturation vapor pressure of methanol and *S* = 0.89 is its gas-phase saturation ratio. This gas-phase saturation ratio was set based on the estimates of the droplet residence time, determined by the initial droplet velocity and the distance between the paper tip and the MS inlet cone, to achieve controlled reduction of the droplet diameter from its initial value to the desired final size.^42^ For a fixed travel time, lower saturation ratios lead to greater evaporative shrinkage and thus smaller final droplet diameters at the MS inlet.

Since the droplet volume was shrinking with time, species were tracked as moles and converted to concentrations using the instantaneous droplet volume, *V*(*t*) = π*D* ^3^(*t*)/6. The full set of coupled differential equations for all chemical species and the droplet diameter were solved in Python using SciPy’s Radau stiff integrator ( *r*_tol_ = 2 × 10^–13^, *A*_tol_ = 1 × 10^–30^).

### Ambient Air Sampling

Sampling was carried out from March 22 to April 1, 2026, over a 10-day period at the Rutgers University–Newark campus, on the roof of Warren Hall (110 Warren St; Lat. 40°44′24″ N, Lon. 74°10′39″ W). The air sampling setup consisted of a filter holder (Savillex, 25 mm single-stage filter assembly) loaded with Whatman P8 filter paper cut to a 1-in. diameter circle and connected via a critical orifice (1 L min^–1^ choked flow) to a diaphragm pump (Gast, model DOA-P104-AA). Air quality data, including concentrations of NO, NO_2_, NO_*x*_, O_3_, PM_2.5_, temperature, relative humidity, precipitation, wind direction, and wind speed were obtained at 6 h intervals from the New Jersey Air Monitoring Network, as summarized in Table S3.

## Results and Discussion

### Speciation Experiments

Ideally, spiking an HgBr_2_ sample with solution containing halide ion L^–^ should yield straightforward ligand addition, forming mixed-halide complexes of the type HgBr_2_L^–^.

R4 $${\mathrm{HgBr}}_{2}+L^{-}⇌{\mathrm{HgBr}}_{2}L^{-}$$

This expectation follows from the strong affinity of Hg­(II) for soft Lewis bases. The extent of complexation is strongly halide-dependent, with prior studies showing that iodide forms significantly more stable complexes (*K*(HgI_2_ + I^–^) = 1.8 × 10^5^ L mol^–1^) than chloride (*K*(HgCl_2_ + Cl^–^) = 19 L mol^–1^) because of the higher polarizability and stronger coordination of I^–^ toward Hg­(II).^41^ However, in our experiments, the monoaddition product often appears only in small amounts, especially in the presence of excess iodide, as the large stability constants of mercuric ions with the iodide drive the reaction network toward the mercuric triiodide ion. Indeed, under the high-reagent conditions (31.5 μM NH_4_I), the mass spectrum is dominated by HgI_3_^–^ (Figure 1c,d), with no observable signals that can be assigned to other mercuric ions, indicating that sequential ligand exchange proceeds nearly to completion. Only at a lower iodide concentration (3.15 μM), HgBr_2_I^–^ becomes observable, along with other halide-exchange ionic species HgBr_3_^–^ and HgBrI_2_^–^ (Figure 1e). To isolate spectra of these ions, background contributions must be removed by subtracting the spectrum obtained during the last elution cycle in the presence of an excess iodide that converts all these species to HgI_3_^–^.

Based on the detected ion intermediates, the iodide-driven transformation can be described by the following stepwise sequence:

R5 $${\mathrm{HgBr}}_{2}+I^{-}⇌{\mathrm{HgBr}}_{2}I^{-}$$

R6 $${\mathrm{HgBr}}_{2}I^{-}⇌\mathrm{HgBrI}+{\mathrm{Br}}^{-}$$

R7 $$\mathrm{HgBrI}+I^{-}⇌{\mathrm{HgBrI}}_{2}^{-}$$

R8 $${\mathrm{HgBrI}}_{2}^{-}⇌{\mathrm{HgI}}_{2}+{\mathrm{Br}}^{-}$$

R9 $${\mathrm{HgI}}_{2}+I^{-}⇌{\mathrm{HgI}}_{3}^{-}$$

Doubly charged HgI_4_^2–^ is never observed under our experimental conditions because of insufficiently high iodide concentration, but bromide ion released in reactions 11 and 13 can react with HgBr_2_ to form HgBr_3_^–^

R10 $${\mathrm{HgBr}}_{2}+{\mathrm{Br}}^{-}⇌{\mathrm{HgBr}}_{3}^{-}$$

Together, these equilibria describe the progressive formation of mixed-halide anions and the strong thermodynamic preference for HgI_3_^–^ under iodide-rich conditions. Direct ligand redistribution between neutral mercuric halides (e.g., HgBr_2_ + HgI_2_ ⇌ 2 HgBrI), which has been observed in solutions and on surfaces,^43–45^ may also occur when the concentrations of the neutrals become sufficiently high but is not required to account for the observed speciation trends. To further substantiate the above halogen-driven transformation mechanism, we employed two complementary strategies. First, high-resolution mass spectrometry was applied to study the evolution in the concentrations of different ionic species directly during each elution without the need of the spectral subtraction. Second, chloride was used in place of iodide, exploiting the fact that its equilibrium constant for complexation with Hg­(II) is approximately 4 orders of magnitude smaller than for iodide.^41^

Using high-resolution mass spectrometry, intermediate halide-exchange ionic species are clearly resolved (Figure 2). Notably, the high-resolution MS enables detection at a substantially lower mercury loading (3.6 ng) than the low-resolution platform (14.4 ng), using a lower iodide concentration (0.16 μM versus 3.15 μM NH_4_I), yet yields the same speciation pattern. This agreement demonstrates that the observed mixed-halide intermediates are intrinsic features of the reaction network rather than artifacts arising from spectral congestion or limited resolving power. The dynamic evolution of this chemistry is illustrated by the total ion current (TIC) and extracted ion current (EIC) measurements shown in Figure 3. Across five sequential elutions, progressive introduction of the iodide reagent leads to its accumulation on the paper substrate, driving a systematic shift in the product distribution toward increasingly iodide-substituted mercury species. During the first elution, the largest contribution to TIC comes from the simple association product-ion HgBr_2_I^–^, followed by HgBr_3_^–^, HgBr_2_I^–^, and HgI_3_^–^. During the third elution, HgBr_2_I^–^ is overtaken by HgBrI_2_^–^, which nevertheless remains the dominant ion even during the fifth elution. This temporal progression is fully consistent with a stepwise halide-exchange process occurring within the paper spray environment. Upon replacing iodide with chloride as the ionization reagent (59.8 μM NH_4_Cl), the same stepwise halide-exchange sequence is observed (Figure 4), represented by HgBr_2_Cl^–^, HgBrCl_2_^–^, and HgBr_3_^–^. High-resolution mass spectra (Figure 5) acquired using a lower HgBr_2_ loading (3.6 ng versus 14.4 ng), but at an identical chloride concentration, allow the resolution of an addditional ion, HgCl_3_^–^. Overall, these high-resolution mass spectra exhibit product distributions consistent with the low-resolution spectra despite differences in mercury loading, further supporting the robustness of the halide-exchange mechanism across platforms and selected experimental conditions.

**Figure 2:**
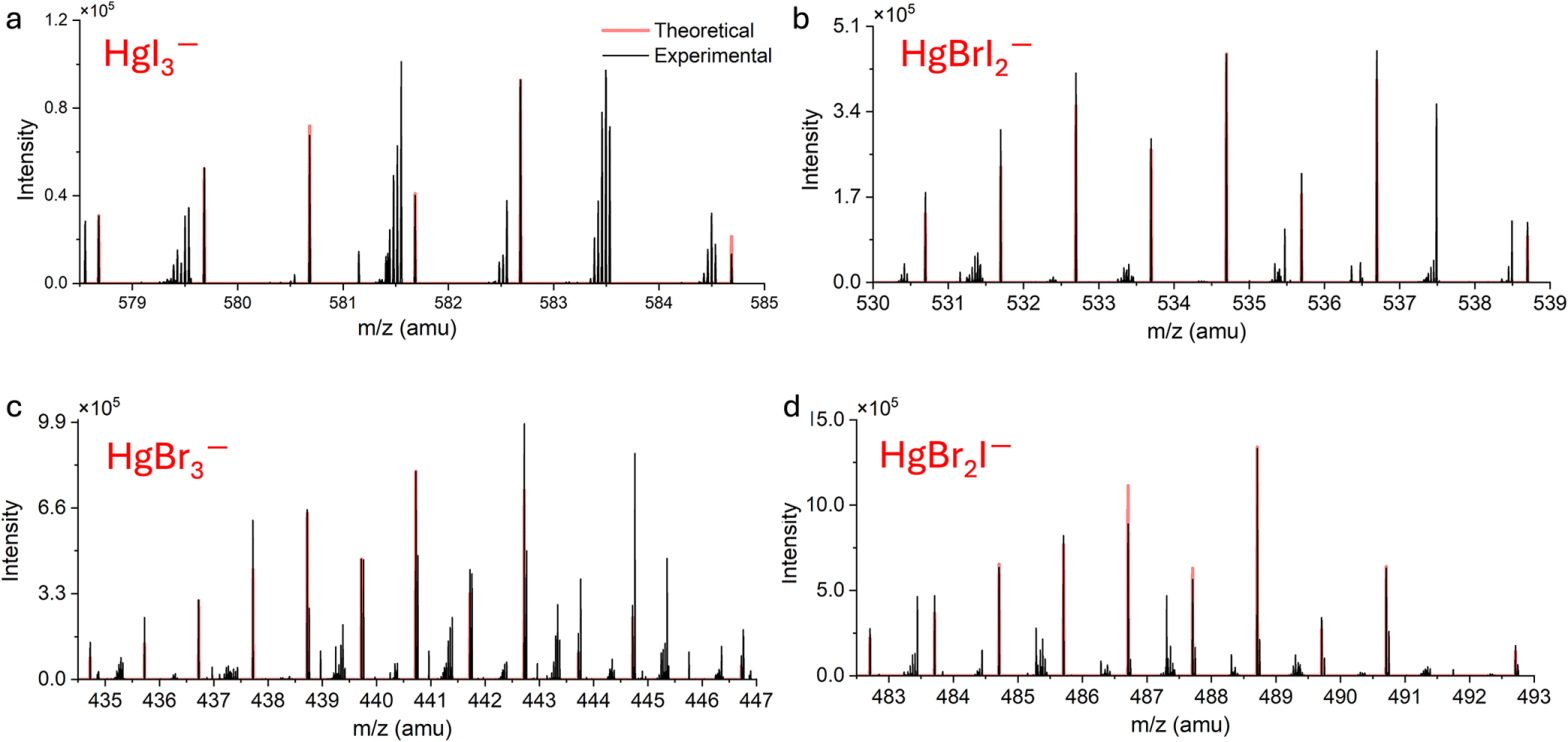
High-resolution mass spectra of 3.6 ng HgBr_2_ loaded on paper and eluted with 0.16 μM NH_4_I: (a) HgI_3_^–^, (b) HgBrI_2_^–^, (c) HgBr_2_I^–^, and (d) HgBr_3_^–^. The mass spectra are obtained during the first elution step.

**Figure 3:**
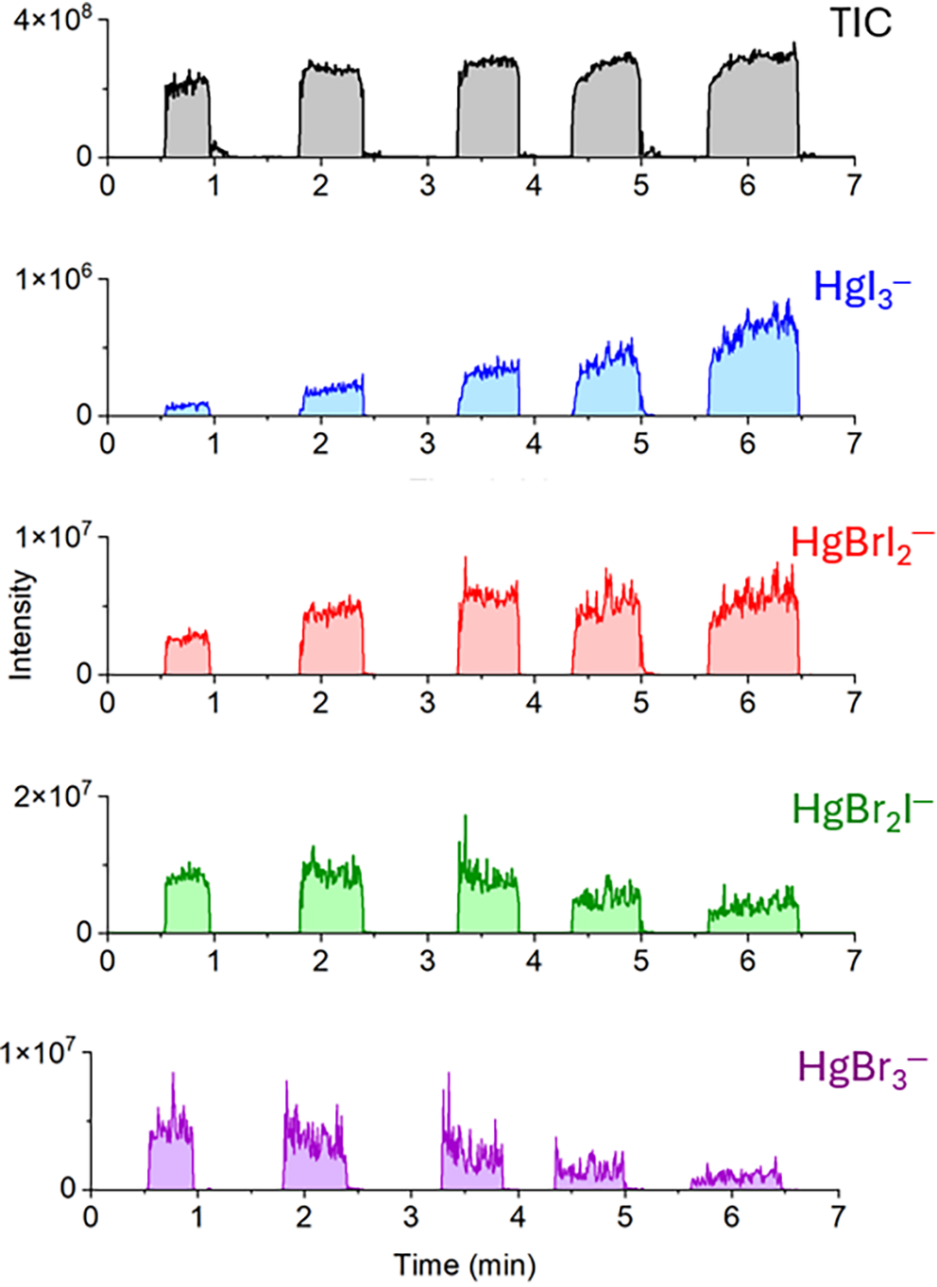
Time evolution of the total ion signal (TIC) and extracted ion signals (EICs) showing iodide-mediated speciation of mercury. Traces for HgI_3_^–^, HgBrI_2_^–^, HgBr_2_I^–^, and HgBr_3_^–^ are displayed over five elution steps. Experimental conditions are the same as in Figure 2.

**Figure 4:**
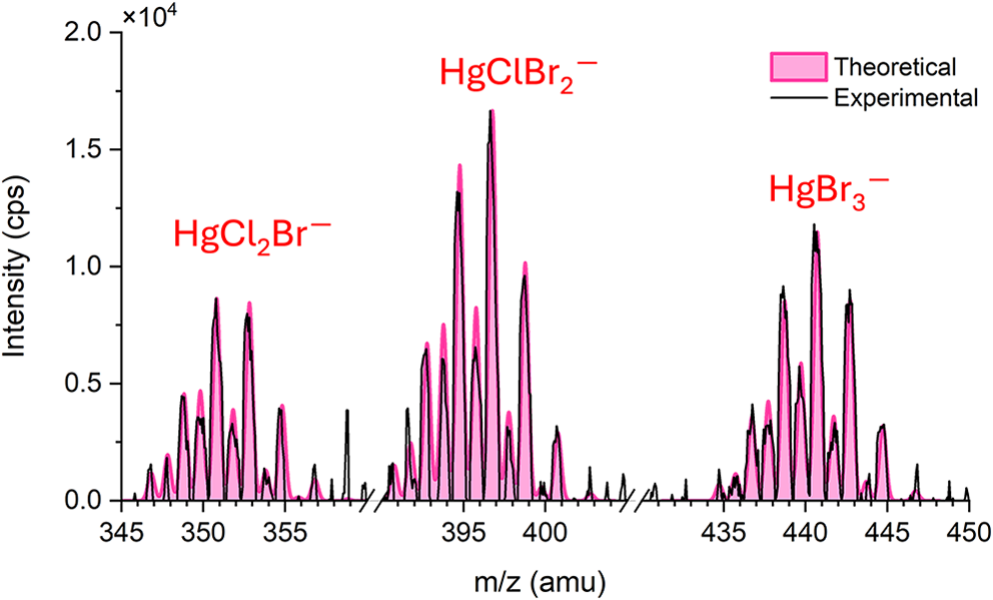
Low-resolution mass spectra obtained with 14.4 ng HgBr_2_ loaded on paper during the first elution with 59.8 μM NH_4_Cl. Background was removed by subtracting the spectrum obtained in a later elution using an excess of iodide to fully convert all species to HgI_3_^–^.

**Figure 5:**
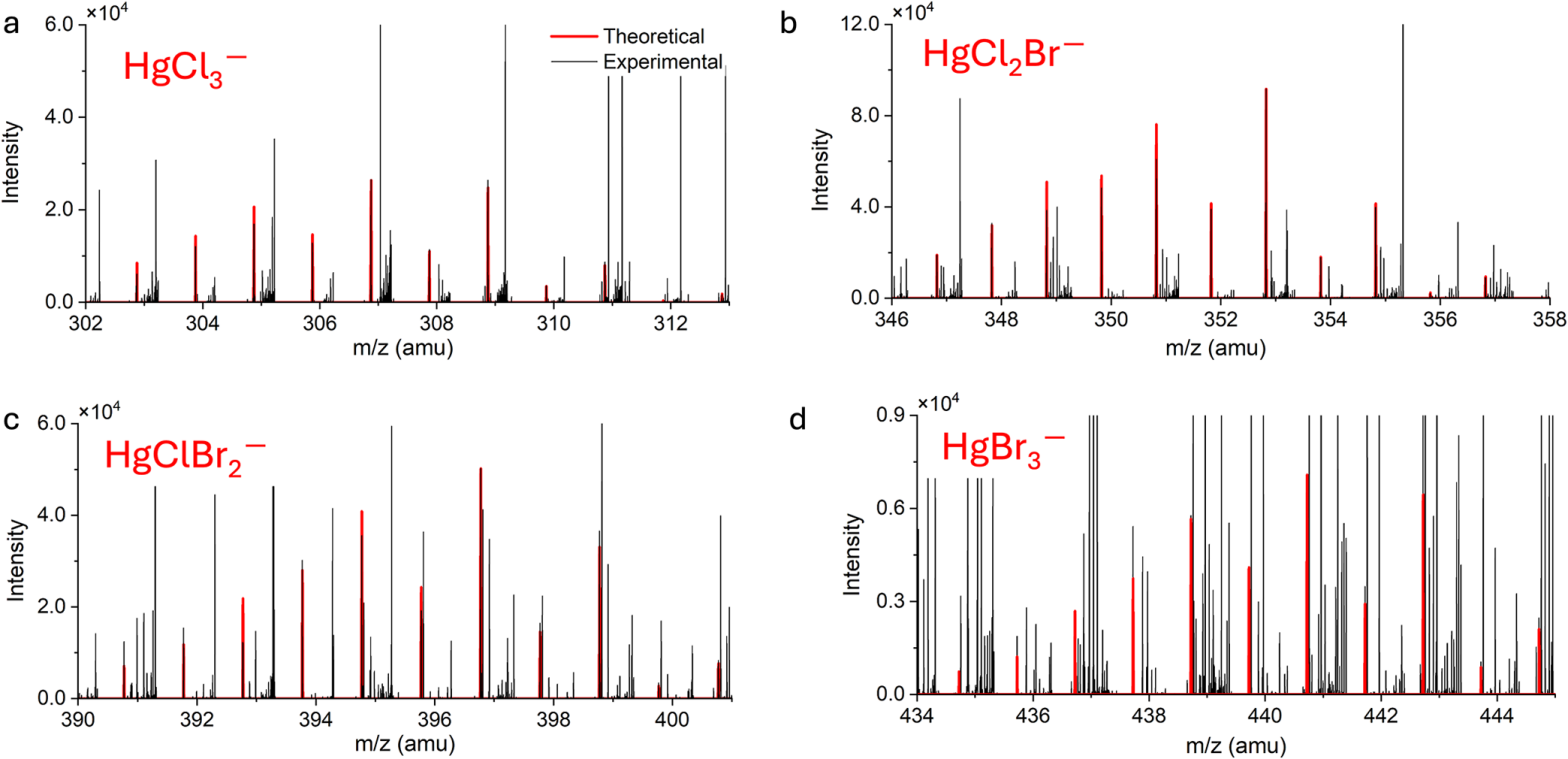
High-resolution mass spectra of 3.6 ng HgBr_2_ loaded on paper and eluted with 59.8 μM NH_4_Cl: (a) HgCl_3_^–^, (b) HgBrCl_2_^–^, (c) HgBr_2_Cl^–^, and (d) HgBr_3_^–^. The mass spectra are obtained during the first elution step.

Our findings differ significantly from those reported in the prior work by Griffiths and Anderson,^46^ who proposed the reaction of I^–^ with HgBr_2_ to proceed as concerted Br^–^ replacement by I^–^ to form a mixed-halide neutral product HgBrI, followed by further such replacement to yield HgI_2_, and eventually, iodide-rich homohalide ion HgI_3_^–^. Only the reaction of Cl^–^ with HgBr_2_ was interpreted as direct ligand association to form a distinct ionic intermediate HgBr_2_Cl^–^ in that study. Because those experiments were conducted at very large halide-to-mercury ratios required for spectroscopic isolation, mixed-halide ionic intermediates HgBrCl_2_^–^ and HgBrI_2_^–^ were neither observed nor considered chemically accessible. In the absence of experimentally detectable intermediates, two distinct mechanistic descriptions for halide substitution had to be invoked, depending on whether the incoming halide was more electronegative (Cl^–^) or less electronegative (I^–^) than bromide. In contrast, by relying on mass spectrometry, we directly detect mixed-halide ionic intermediates for both iodide- and chloride-driven exchange, including species such as HgBrCl_2_^–^ and HgBrI_2_^–^. Observation of these intermediates supports a unified mechanistic framework in which stepwise ligand exchange proceeds through reversible halide addition and dissociation, rather than through separate pathways of either addition or replacement, depending on electronegativity of reacting halide ion. The distribution of final detected products is governed by the molar ratio of the initial mercuric bromide to the reagent halide ion (Cl^–^ or I^–^), as well as by the relative thermodynamic stabilities of the resulting complexes.

To demonstrate the applicability of our method beyond mercuric bromide, we speciated HgCl_2_ and HgI_2_, using NH_4_I and NH_4_Cl as ionizing reagents. Figure 6 shows that in both systems only two ions, HgI_3_^–^ and HgClI_2_^–^, are detected by the low-resolution MS. Similar to HgBr_2_, these ions are a result of stepwise halide addition and loss reactions, but the equilibrium is shifted toward HgI_3_^–^ due to the 4 orders of magnitude higher stability constant in mercuric iodide ions relative to corresponding chlorides. The intermediate ions may become detectable by the high-resolution MS or when using NH_4_Br as the ionizing reagent. Bromide might be the most suitable reagent for the speciation of HgCl_2_ and HgI_2_ based on its stability constant, which is within approximately 2 orders of magnitude between those of chloride and iodide (Table S1).

**Figure 6:**
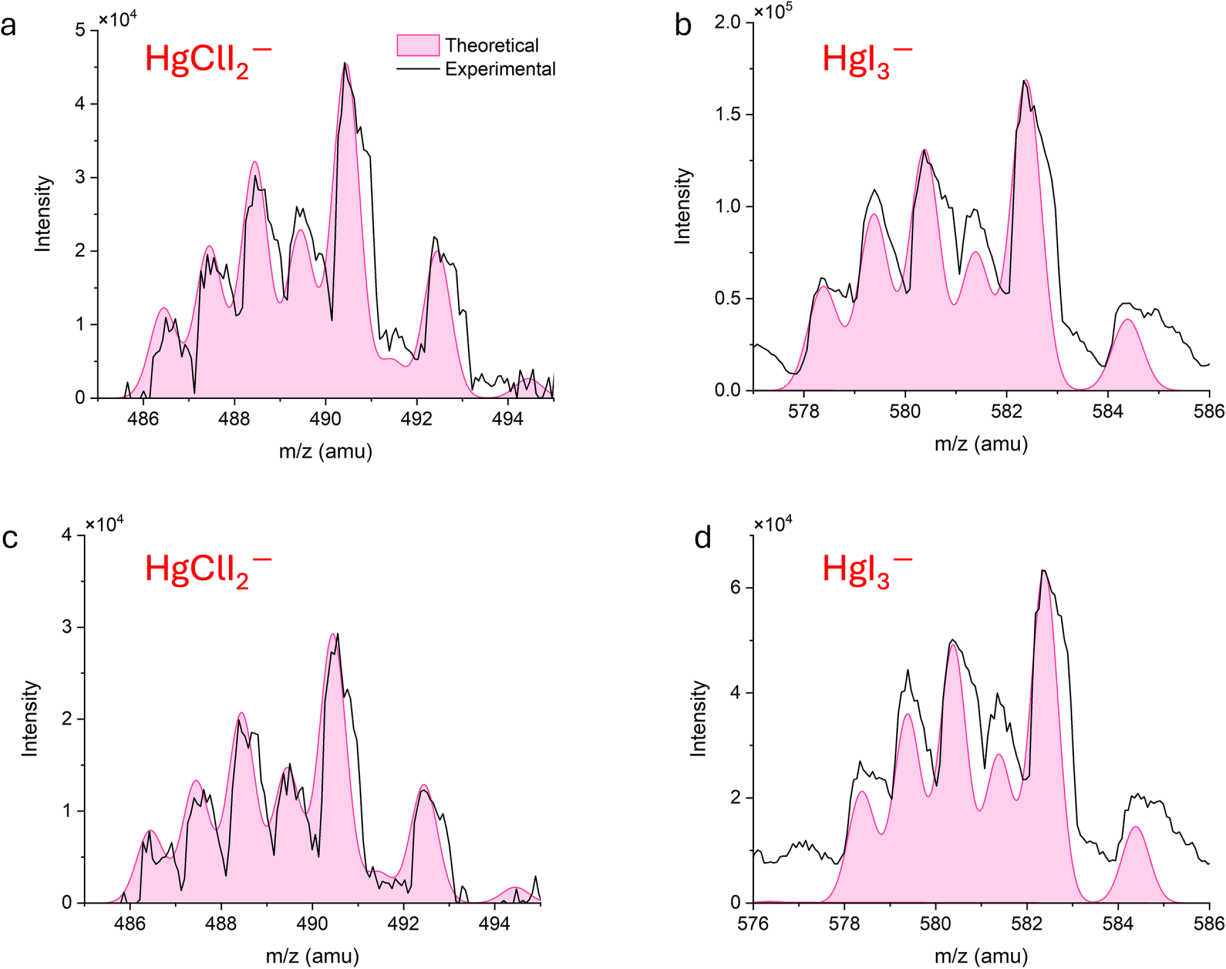
Low-resolution mass spectra illustrating speciation of HgCl_2_ and HgI_2_: Panels (a) and (b) show HgClI_2_^–^ and HgI_3_^–^ generated from 10.6 ng HgCl_2_ with 1.05 μM NH_4_I. Panels (c) and (d) show the same products, but generated from 17.5 ng HgI_2_ in the presence of 300 μM NH_4_Cl.

### Kinetic Model of Spray Droplet Chemistry

A kinetic model was developed to evaluate whether the proposed halide-exchange mechanism can reproduce the experimentally observed evolution of mercury–halide species during paper spray ionization. The model is designed to test mechanistic consistency rather than to determine absolute species concentrations, with emphasis placed on capturing trends in speciation as a function of the relative concentrations of the two reagent ions (Cl^–^ and I^–^) and the resulting chemistry within evaporating droplets.

Two iodide concentrations, low ([NH_4_I^–^] = 0.16 μM) and high ([NH_4_I^–^] = 26.2 μM), were simulated for iodide-mediated exchange, and a single high-chloride condition ([NH_4_Cl^–^] = 59.8 μM) was simulated for chloride-mediated exchange (Figure 7). With the reactants still on the paper filter, the ionized fraction is about 0.01% under the low iodide condition (Figure S1b), but under the high iodide condition, 84% of HgBr_2_ is converted to HgI_3_^–^—the dominant ion (Figure S1a). With 59.8 μM chloride, only 0.1% of mercury is present in the ionic form on the filter (Figure S1c). The ionization increases drastically in the evaporating spray droplets, which over 1.2 ms shrink in size 4-fold (Figure 1b), corresponding to a 64-fold decrease in the droplet volume and associated 64-fold increase in the concentrations of the dissolved neutrals and ions. Under high iodide, the model predicts near-complete conversion of HgBr_2_ to HgI_3_^–^, with approximately 99% of mercury present as HgI_3_^–^ (Figure 7a). This predicted speciation is consistent with the experimentally observed mass spectrum shown in Figure 1c,d. Under low iodide, the kinetic model predicts comparable concentrations of multiple mercury–halide species within the droplet phase (Figure 7b), indicating that all species should, in principle, be detectable, in agreement with the mass spectrometry experiment (Figure 2). Although the model overpredicts the abundance of HgBr_3_^–^ relative to the experimental mass spectrum, this discrepancy is expected, as the kinetic model describes chemical evolution within evaporating droplets and does not explicitly account for ion ejection efficiency or differential detection sensitivity in the mass spectrometer. Previous studies have shown that ion distributions within droplets are not uniform and depend strongly on the ion size and charge density, with bulkier ions of equal charge preferentially residing near the droplet interface and exhibiting enhanced MS sensitivity due to more efficient ejection.^47,48^ In the HgBr_2_ + I^–^ system, HgBr_3_^–^ is the smallest anion among the detected charged mercury complexes and therefore has the highest charge density. As a result, its reduced signal intensity relative to bulkier species is consistent with established droplet–ion partitioning behavior, explaining its apparent underrepresentation in the experimental spectrum and its role as the sole outlier between modeled and observed speciation.

**Figure 7:**
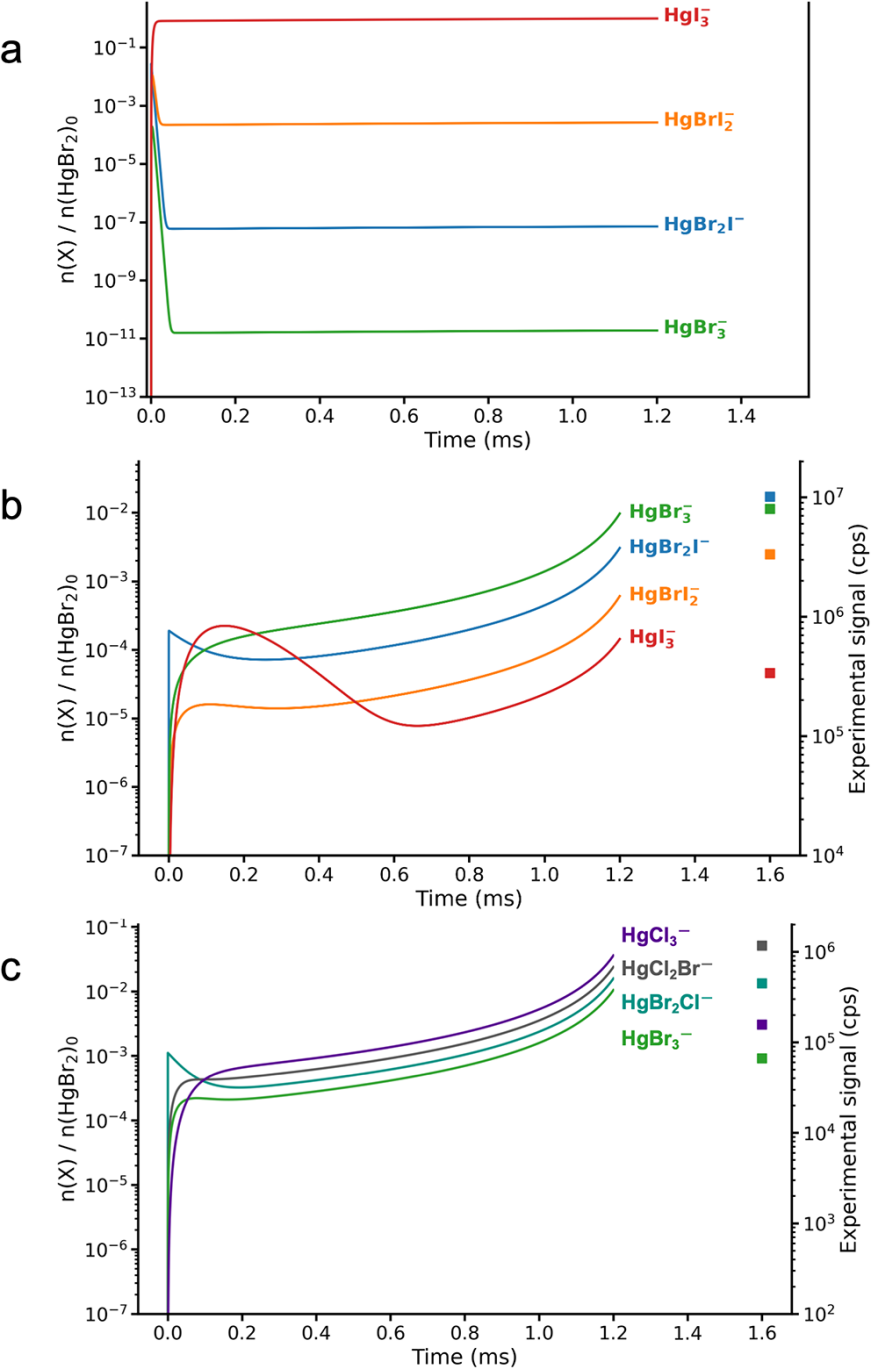
Kinetic-model predictions of halide-mediated speciation pathways for 3.6 ng HgBr_2_ under three reagent-ion conditions: (a) 26.2 μM NH_4_I, (b) 0.16 μM NH_4_I, and (c) 59.8 μM NH_4_Cl. The primary *y*-axis represents the normalized mole fractions of different species relative to the initial HgBr_2_ amount, *n*(X)/n­(HgBr_2_)_0_. The secondary *y*-axis shows the signals of those species measured by high-resolution MS under identical to model conditions

Figure 7c corresponds to the modeled high-chloride condition, selected to reflect the chloride speciation experiments. Similar to the low iodide case, the kinetic model predicts comparable concentrations of the mercury–halide complexes, indicating that all species are expected to be detectable by mass spectrometry. This prediction is consistent with the experimentally observed speciation patterns in both the high- and low-resolution spectra (Figure 5). The sole discrepancy between modeled and experimental speciation is associated with HgCl_3_^–^. This species is the smallest anionic mercury complex and therefore possesses the highest charge density among the detected ions. Its reduced signal intensity relative to bulkier complexes is thus consistent with established droplet–ion partitioning and ejection behavior, rather than a failure of the proposed reaction mechanism.

An important outcome of this modeling exercise is that HgBr_2_ does not react with 0.16 μM NH_4_I while on the paper filter due to kinetic and thermodynamic limitations imposed by the low concentrations of both chemicals, with ionized fraction of only 10^–6^–10^–4^ (Figure S1b). In the evaporating spray droplets, this fraction rises sharply to 10^–4^–10^–2^, driven by the significant increase in the concentrations of all species (Figure 7b). A large fraction of the ionic species retain their original ligand (Br), either fully or at least partially, allowing the composition of the original neutral species (HgBr_2_) to be inferred. Hence, the 0.16 μM iodide concentration represents a Goldilocks zone where the collected filter sample would remain mostly unperturbed, yet sufficient ionization can be achieved in spray droplets, making it possible not only to detect HgBr_2_ but also retrieve its original speciation. We envision that a speciation scheme can be developed where a filter with collected GOM sample is cut into several pieces and each piece is analyzed using different reagent ions (Cl^–^, Br^–^, and I^–^) present in different concentrations. The resulting ion distributions can be processed using a model, such as this kinetic model or a machine learning model, to reconstitute the original sample composition.

### Limit of Detection

Quantitative performance of the RPS-MS platform was assessed through calibration experiments in which iodide was introduced at sufficiently high concentrations to drive complete conversion of the loaded HgBr_2_ to HgI_3_^–^. This ensured that only a single, well-defined ion was produced, enabling straightforward quantification without contributions from intermediate mixed-halide species and also prevented signal partitioning among multiple complexes, thereby improving the overall signal-to-noise ratio.

The calibration was performed for the low-resolution and high-resolution instruments. Both platforms produced well-behaved calibration curves, as shown in Figure 8. The limits of detection obtained from these calibration curves were 306 and 70 pg for the low- and high-resolution systems, respectively. The detection limit obtained with the high-resolution system is much lower than typical reactive mercury loadings in ambient samples (0.4–3.5 ng; Table S2),^22,49,50^ demonstrating that the method provides sufficient sensitivity for quantitative measurements of mercuric halides collected under real-world conditions. Assuming a two-week sampling period at a 1 LPM flow, the high-resolution LOD value corresponds to an atmospheric concentration of 27.8 pg m^–3^. This LOD is in the range of values reported for other mass spectrometric methods, including 90 pg for electron impact ionization MS^17^ and 4–11 pg for an atmospheric pressure chemical ionization MS,^18^ but it is higher than the 0.5 pg detection limit reported for the Tekran 2537X (Tekran Corporation, Toronto, Canada), which uses cold vapor atomic fluorescence for the detection of Hg­(II) after its conversion to Hg(0).

**Figure 8:**
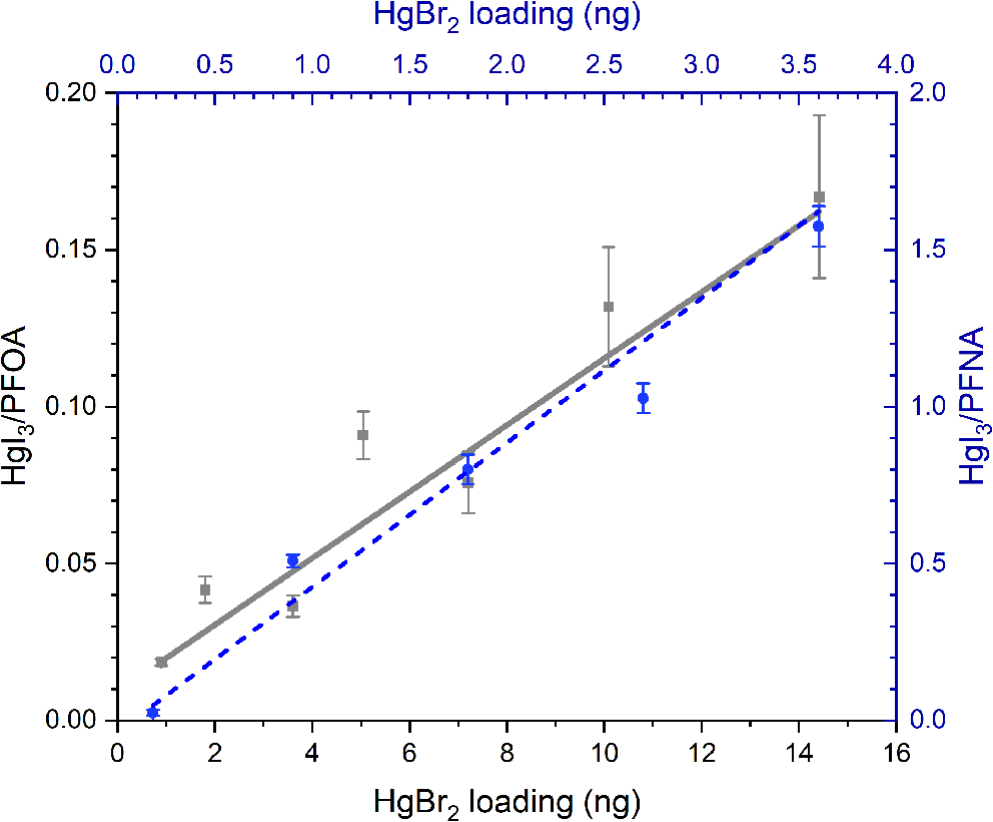
Calibration curves for HgBr_2_ obtained using low- and high-resolution mass spectrometry under iodide-assisted ionization conditions. Gray squares (left axis) show the HgI_3_^–^/PFOA response measured by the low-resolution MS at 31.5 μM NH_4_I. Blue circles (right axis) show the HgI_3_^–^/PFNA response measured by the high-resolution MS at 26.2 μM NH_4_I. Solid and dashed lines represent the linear fits to the low- and high-resolution data sets, respectively. Error bars denote one standard deviation from three replicate measurements. The slopes of the calibration curves for the low- and high-resolution systems are (1.08 ± 0.13) × 10^–2^ and 0.43 ± 0.04, with corresponding *R* ^2^ values of 0.93 and 0.97.

### Ambient Reactive Mercury Analysis and Application to Other Metals

Before collecting ambient samples, we evaluated the matrix effects on RPS-MS, using succinic acid (C_4_H_6_O_4_) and ammonium sulfate ((NH_4_)_2_SO_4_) to represent organic and inorganic aerosol constituents, respectively. Two loading conditions were chosen to represent urban atmospheres with moderate and high particulate matter levels. For the moderately polluted environment, 22.5 μg of succinic acid and 18.6 μg of (NH_4_)_2_SO_4_ were loaded on a filter paper triangle (1/8 of a 25 mm diameter filter) from an aqueous solution, corresponding to air containing 22.8 μg m^–3^ PM2.5 sampled at 1 L per minute over 10 days. For the heavily polluted urban environment, the loaded amounts were increased 10 times to represent 228 μg m^–3^ PM2.5. In both cases, after drying the matrix solution, the triangle was spiked with 14.4 ng HgBr_2_, redried, and analyzed using the high concentration of ammonium iodide to fully convert HgBr_2_ to HgI_3_^–^. As shown in Figure S3, the signal intensity decreased by a factor of 3 under moderate-matrix conditions and by approximately 1 order of magnitude under high-matrix conditions relative to clean paper. Nevertheless, HgI_3_^–^ remained clearly detectable in both cases. These results demonstrate that RPS-MS is applicable for analysis of mercuric halides in ambient air even without dedicated matrix-effect mitigation strategies, including conditions of severe air pollution. In the future, the sampling technique can be modified to minimize the amount of particulate matter collected on the filter, or sample post processing, such as desalting^30^ and electrokinetic manipulation,^51^ can be employed to reduce the matrix effect.

We selected cellulose paper as a filtering medium for collecting ambient samples because it traps gaseous HgCl_2_ with high efficiency, similar to sodium chloride and fully deprotonated dicarboxylic acids,^52,53^ while also providing strong and stable spray performance during RPS-MS analysis.^54^ However, a systematic evaluation of other substrates with different physicochemical properties will be an important subject for future work. Since no PTFE prefilter was used in front of the cellulose filter to remove airborne particulate matter, our sample consisted of both GOM and PBM. After 10 days of collection, particulate matter was clearly noticeable as a gray discoloration of the filter paper (Figure S4). After sample collection, the filter was cut into eight pieces with sharp scissors for use in speciation and quantification analyses (Figure S4).

During speciation, the paper was eluted with 0.15 μM NH_4_I. This concentration was selected to strike a balance between minimizing exchange and ensuring sufficient sensitivity. No mercuric species were detected during the first elution, whereas the second elution showed the highest mercuric signals (Figure 9), likely indicating that the first elution transported the collected GOM species closer to the paper tip. Even at this low iodide concentration, most of the detected mercury (78%—based on the signal intensity during the first elution) was present as HgI_3_^–^, followed by HgClI_2_^–^ (16%) and HgBrI_2_^–^ (6%). The observation of the latter two ions points to the presence of chlorine-containing and bromine-containing mercuric compounds corresponding to ClHgX and BrHgX compositions, where X could be a halogen or some other ligand. Knowing that HgBr_2_ undergoes less halide exchange during ionization than HgCl_2_ because of the higher stability constant for the mercuric complexes with bromide, we may conclude that the ClHgX-to-BrHgX loading ratio in the sample is higher than the corresponding HgClI_2_^–^-to-HgBrI_2_^–^ signal ratio. The absence of ions corresponding to oxygen-containing and nitrogen-containing species proposed by theoretical studies^11,12^ can be attributed to two factors. First, halides exhibit a stronger affinity for mercury than other ligands and are more stable during exchange reactions that occur even at low iodide concentrations, leading to the loss of the nonhalogen ligand and the formation of HgBrI_2_^–^ (Figure S2). Second, the concentrations of these species are likely lower than those of mercuric halides, consistent with the recent findings of Jokinen et al.,^15^ who reported simple and mixed halides as the only mercuric species detected in direct atmospheric measurements of oxidized mercury in polar regions, using nitrate chemical ionization mass spectrometry. Modeling studies also suggest that HgCl_2_ may represent nearly half of atmospheric GOM, being formed via aerosol processing of the oxygen-containing Hg­(II).^7^

**Figure 9:**
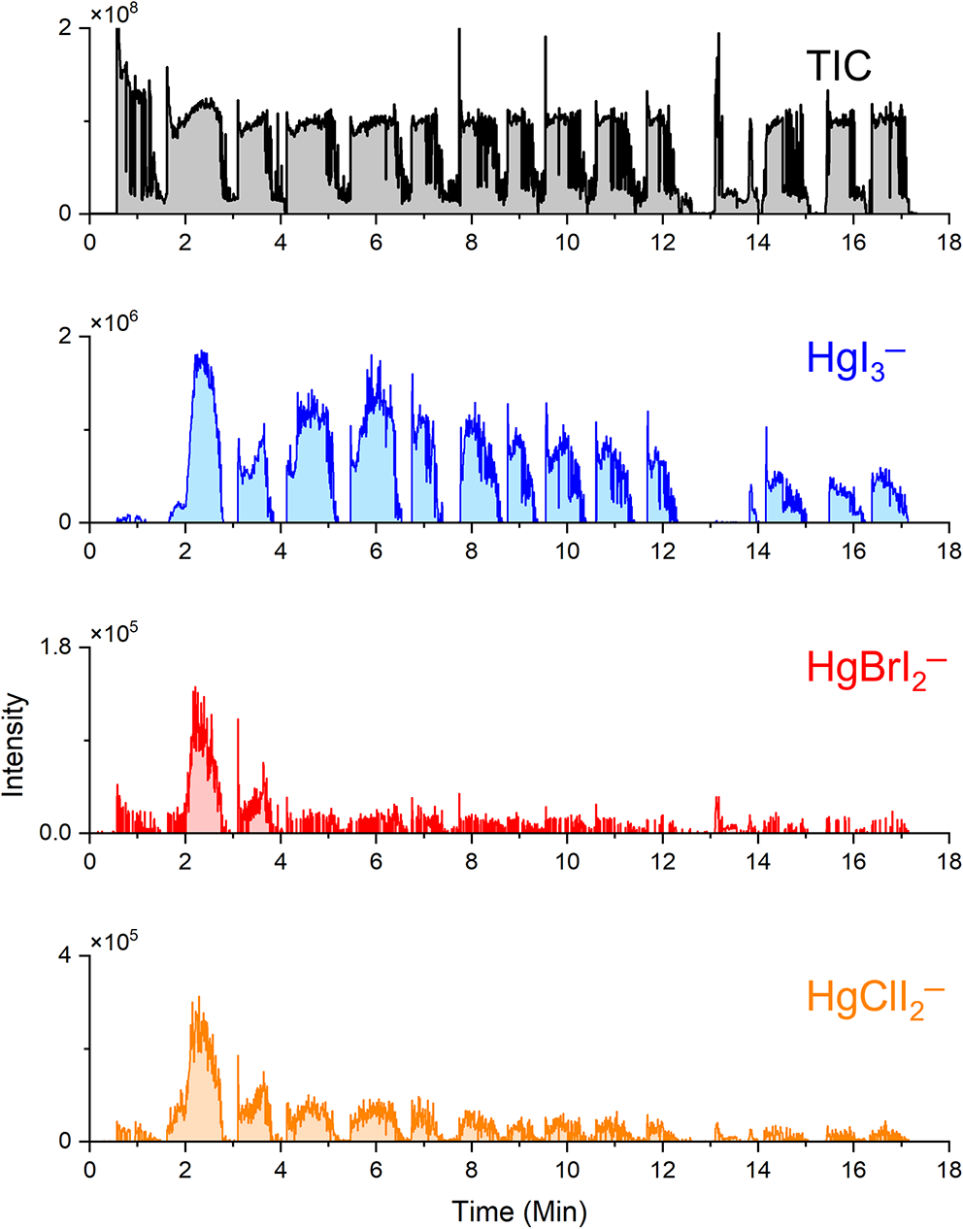
Time evolution of the total ion signal (TIC) and extracted ion signals (EICs), showing iodide-mediated speciation of atmospheric mercury in a sample collected on a paper filter over 10 days. Traces correspond to HgI_3_^–^, HgBrI_2_^–^, and HgClI_2_^–^. The concentration of the ionizing reagent NH_4_I is 0.15 μM.

During quantification, the sample was eluted using a significantly higher NH_4_I concentration (34.29 μM) to convert all mercuric species to HgI_3_^–^. Similar to the calibration experiments, 12.5 ng PFNA was added to the sample as an internal standard. The PFNA signal decreased by a factor of 10 relative to that of a clean filter because of matrix effects. Based on Table S3, the average PM_2.5_ concentration was 7.1 μg m^–3^. For a 10-day collection period, this corresponds to 12.8 μg of matrix deposited on one triangular paper sector. According to the calibration curve, such filter sector contained 0.51 ng RM, corresponding to 4.06 ng on the entire filter. Using the sampling flow rate and sampling duration, the average ambient RM concentration was estimated as 286 ± 28 pg m^–3^. This value agrees with RM concentrations reported for other urban areas 12–660 pg m^–3^,^55^ being at a higher range likely because our sampling location is near several major emission sources of elemental and oxidized mercury, including a municipal waste incineration plant (3.5 km), Newark Liberty International Airport (4 km), Port Newark–Elizabeth Marine Terminal (4.5 km), and legacy mercury contamination sites in the Hackensack River estuary (10–15 km).^56^

To examine the applicability of our method toward other metals, we analyzed 23.2 ng CdCl_2_ loaded on a paper triangle from solution and eluted using 22.46 μM NH_4_I as the ionizing reagent. Figure 10 shows all possible exchange ion products, including CdCl_3_^–^, CdCl_2_I^–^, CdClI_2_^–^, and CdI_3_^–^, demonstrating the successful speciation of CdCl_2_ by iodide RPS-MS. It is noted that a higher iodide concentration was required in this analysis because of the significantly lower stability constants for complexes of halides with cadmium than with mercury.^57^

**Figure 10:**
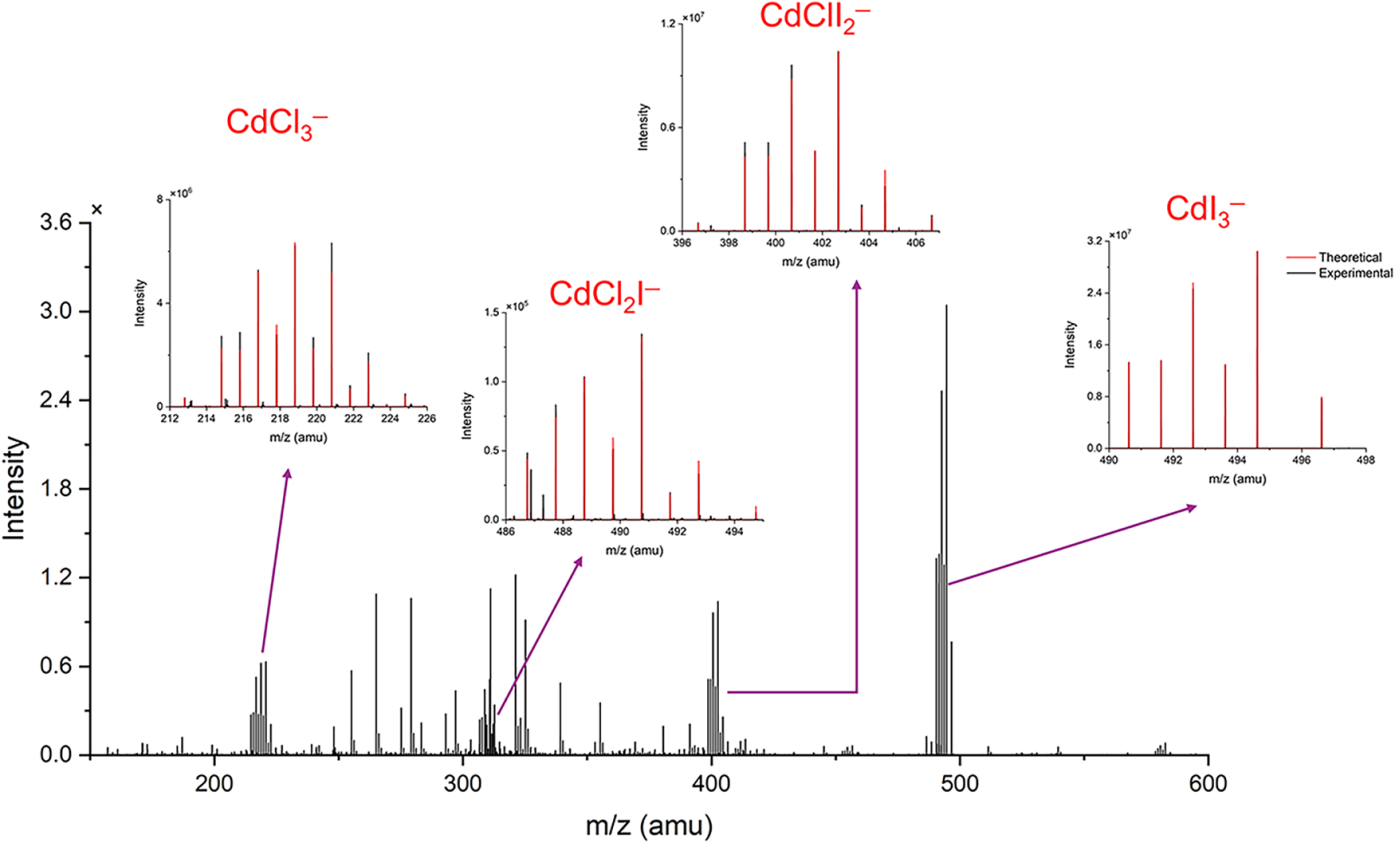
Speciation of 23.2 ng CdCl_2_ eluted with 22.46 μM NH_4_I. The high-resolution mass spectrum, obtained during the first elution, shows the presence of CdI_3_^–^, CdClI_2_^–^, CdCl_2_I^–^, and CdCl_3_^–^.

## Conclusions

In this work, we demonstrate that reactive paper spray mass spectrometry provides a nonthermal, reagent-directed strategy for probing the native chemical speciation of gaseous oxidized mercury at trace levels. Using HgCl_2_, HgBr_2_, and HgI_2_ as chemically relevant surrogates for gaseous oxidized mercury along with chloride and iodide ions as reagents, we show that ionization proceeds through reversible halide addition and halide loss steps, giving rise to a network of mixed-halide ionic intermediates that were previously inaccessible by experimental measurements relying on electronic absorption spectroscopy.

By comparing iodide- and chloride-mediated chemistry, we establish that the extent of ligand exchange is governed by the relative thermodynamic stability of the resulting Hg­(II)–halide complexes and halide concentration. Iodide drives progressively deeper halide substitution through strongly favorable exchange equilibria, leading to near-quantitative formation of HgI_3_^–^ under iodide-rich conditions. Using iodide, even at lowera lower concentration, concentration, allows achieving a significantly higher degree of ionization compared to chloride, with only moderate overexchange. Direct detection of mixed-halide ionic intermediates under comparable concentrations of mercuric species and the ionizingreagent supports a unified mechanistic framework in which mercuric halide complexation proceeds through reversible, stepwise ligand-exchange rather than the previously postulated distinct addition versus replacement pathways controlled by the relative electronegativity of the halide reagents involved.

Kinetic modeling of chemistry occurring within evaporating spray microdroplets reproduces the experimentally observed speciation trends across explored reagent-ion conditions and provides mechanistic consistency between observed intermediates and the proposed reaction network. Quantitative calibration performed under iodide-rich conditions further demonstrates that reagent-controlled chemistry can be exploited to generate a single dominant product ion, HgI_3_^–^, enabling sensitive and reproducible quantification of mercuric halides at loadings relevant to atmospheric GOM samples. On the other hand, using low iodide during analysis allows retrieval of the native sample speciation.

The applicability of the method was further validated through ambient air sampling and extended to cadmium as another oxidized metal. Ambient air sampling yielded an ambient reactive mercury concentration of 286 ± 28 pg m^–3^ in Newark, NJ, that contained BrHgX and ClHgX (where X could be a halide or a nonhalide ligand).

Together, these results establish RPS-MS as a chemically tunable platform for accessing the ligand-exchange chemistry of oxidized mercury species under ambient ionization conditions. More broadly, this reagent-directed, nonthermal approach provides a generalizable framework for interrogating the intrinsic speciation and overall concentration of ultratrace oxidized metal systems that might be inaccessible to conventional analytical methods.

## Supplementary Material



## Notes

Kinetic parameters and diffusion coefficients for halide-mediated complexation of mercuric halides; literature-derived reactive mercury (RM) concentrations and estimated collected masses; time-resolved air quality data during the field study; kinetic-model predictions of HgBr_2_ speciation under different reagent-ion conditions, including comparison to MS signals; high-resolution mass spectra of atmospheric reactive mercury; evaluation of matrix effects; and images of sampled filter paper (PDF)
