# Supplementary material for: Reactive Paper Spray Mass Spectrometry Enables Speciation of Trace Levels of Mercuric Halides
Source: Anal Chem. 2026 Jun 1;98(23):17453–65. doi: 10.1021/acs.analchem.6c02808 (PMC13276850; doi:10.1021/acs.analchem.6c02808)
Supplement: Supplementary file 1 [file ac6c02808_si_001.pdf]

Supporting Information

Reactive Paper Spray Mass Spectrometry Enables Speciation of Trace Levels of Mercuric Halides

Mohammad Borna Bahramsari[1], Md Tanim-Al Hassan[1], Hao Chen*[1], and Alexei F Khalizov*[1,2]

[1]Department of Chemistry and Environmental Science, New Jersey Institute of Technology, Newark, New Jersey 07102, United States

[2]Department of Chemical and Materials Engineering, New Jersey Institute of Technology, Newark, New Jersey 07102, United States

*Email: Alexei F Khalizov (khalizov@njit.edu)
Hao Chen (hao.chen.2@njit.edu)

Table of Contents

Table S1. Diffusion coefficients and kinetic parameters used to parameterize the stepwise ionic exchange model. S2

Table S2. Reactive mercury (RM) concentrations in ambient air and estimated collected sample masses derived from literature values. S2

Table S3. EPA criteria pollutants and meteorological parameters during ambient air sampling. S2

Figure S1. Kinetic-model predictions of halide-mediated speciation pathways for $HgBr_2$ under different reagent-ion conditions. S4

Figure S2. High-resolution mass spectra of atmospheric reactive mercury collected on paper and eluted with NH_4I. S5

Figure S3. Mass spectra illustrating the influence of matrix effects under different matrix-loading condition. S6

Figure S4. Photograph of sampled paper-filter sectors used for speciation and quantification. S7

Table S1: Diffusion coefficients and kinetic parameters used to parameterize the stepwise ionic exchange model describing the reversible complexation of mercury dihalides with halide anions.

Reaction	$D_1(X^-)^a$	$D_2(HgX_2)^a$	k_{+1}^b	k_{-1}^c	K_{eq}^d
$HgCl_2 + Cl^- \rightleftharpoons HgCl_3^-$	2.2×10^{-9}	9.7×10^{-10}	1.4×10^{10}	7.5×10^{8}	1.9×10^{1}
$HgBr_2 + Br^- \rightleftharpoons HgBr_3^-$	2.0×10^{-9}	9.1×10^{-10}	1.4×10^{10}	1.2×10^{7}	1.2×10^{3}
$HgI_2 + I^- \rightleftharpoons HgI_3^-$	1.9×10^{-9}	8.5×10^{-10}	1.4×10^{10}	7.8×10^{4}	1.8×10^{5}

[a]Diffusion coefficients ($m^2\,s^{-1}$) for the incoming halide (D_1) and neutral mercuric halide (D_2).

[b]Forward rate constants ($M^{-1}\,s^{-1}$).

[c]Reverse rate constants (s^{-1}).

[d]Equilibrium constants defined as $K_{eq} = k_{+1}/k_{-1}$.

Table S2: Reactive mercury (RM) concentrations in ambient air and estimated collected sample masses derived from literature values.

Location	RM ($pg\,m^{-3}$)	Collected RM Mass[a] (ng)	Reference
Mauna Loa, HI, USA	133	2.68	[1]
Sydney, Australia	18	0.36	[2]
Nanjing University, China	160	3.49	[3]

[a] Collected RM mass calculated assuming an air sampling flow rate of 1 L min^{-1} over a 2 week collection period.

Table S3: The EPA criteria pollutants and meteorological parameters during ambient air sampling obtained from the New Jersey Air Monitoring Network [4]. O_3 data were acquired from the Bayonne station, whereas all other parameters were obtained from the Elizabeth Trailer station, the closest monitoring site to NJIT.

Date	Time	NO_2 (ppm)	NO (ppm)	NO_x (ppm)	$PM_{2.5}$ ($\mu g\ m^{-3}$)	O_3 (ppm)	Temp (°F)	RH (%)	Rain (in)
3/22/26	12:00 PM	0.010	0.004	0.015	5.4	0.033	48	66.4	0.001
3/22/26	6:00 PM	0.018	0.006	0.025	13.3	0.034	60	53.7	0
3/22/26	24:00 AM	0.037	0.014	0.053	17.6	0.016	54	72	0.073
3/23/26	6:00 AM	0.018	0.009	0.028	10.7	–	48	77.8	0.302
3/23/26	12:00 PM	0.018	0.017	0.036	2.6	0.020	40	78	0.071
3/23/26	6:00 PM	0.012	0.011	0.024	2.4	0.022	40	66.9	0.008
3/23/26	24:00 AM	0.007	0.002	0.010	1.1	0.033	34	49.6	0
3/24/26	6:00 AM	0.015	0.004	0.020	0.5	0.024	32	52.9	0
3/24/26	12:00 PM	0.015	0.015	0.032	2.2	0.031	36	40.3	0
3/24/26	6:00 PM	0.017	0.018	0.036	6.0	0.037	45	29	0
3/24/26	24:00 AM	0.022	0.014	0.037	1.5	0.029	39	43.8	0
3/25/26	6:00 AM	0.024	0.011	0.036	6.5	–	37	53.8	0
3/25/26	12:00 PM	0.034	0.041	0.076	10.5	–	43	42.8	0
3/25/26	6:00 PM	0.023	0.021	0.045	9.2	–	51	38.8	0
3/25/26	24:00 AM	0.018	0.010	0.029	6.4	–	46	42.5	0
3/26/26	6:00 AM	0.021	0.016	0.039	3.7	–	47	57.6	0
3/26/26	12:00 PM	0.023	0.022	0.046	10.2	–	57	47.8	0.001
3/26/26	6:00 PM	0.017	0.009	0.027	17.9	0.055	74	30.6	0
3/26/26	24:00 AM	0.017	0.004	0.021	18.8	0.045	69	47.5	0.023

Date	Time	NO_2	NO	NO_x	$PM_{2.5}$	O_3	Temp	RH	Rain
3/27/26	6:00 AM	0.014	0.003	0.017	15.8	0.025	61	72	0.002
3/27/26	12:00 PM	0.009	0.006	0.016	1.5	0.031	52	48.6	0
3/27/26	6:00 PM	0.012	0.007	0.020	2.1	0.033	49	37.1	0
3/27/26	24:00 AM	0.015	0.005	0.021	2.8	0.030	43	34.7	0
3/28/26	6:00 AM	0.008	0.002	0.011	0.1	0.032	34	36.5	0
3/28/26	12:00 PM	0.003	0.002	0.006	1.9	0.037	32	31.5	0
3/28/26	6:00 PM	0.002	0.001	0.004	2.2	0.040	41	19.5	0.001
3/28/26	24:00 AM	0.016	0.001	0.018	3.1	0.036	38	25.9	0
3/29/26	6:00 AM	–	–	–	5.1	0.008	34	40.7	0
3/29/26	12:00 PM	0.019	0.028	0.048	5.0	0.030	41	39.2	0
3/29/26	6:00 PM	0.006	0.003	0.010	5.8	0.045	54	22.7	0
3/29/26	24:00 AM	0.015	0.008	0.025	4.3	0.038	48	39.9	0
3/30/26	6:00 AM	0.014	0.009	0.024	4.4	–	47	55.2	0
3/30/26	12:00 PM	0.018	0.020	0.040	7.9	0.027	57	49.2	0
3/30/26	6:00 PM	0.013	0.010	0.024	13.2	0.043	69	32.6	0
3/30/26	24:00 AM	0.013	0.004	0.018	8.7	0.032	65	48.4	0
3/31/26	6:00 AM	0.017	0.009	0.027	9.2	0.023	64	54.9	0.008
3/31/26	12:00 PM	0.018	0.015	0.034	10.5	0.029	69	45.5	0.001
3/31/26	6:00 PM	0.012	0.006	0.019	13.0	0.044	78	37	0.001
3/31/26	24:00 AM	0.013	0.005	0.019	10.3	0.038	74	45	0
4/1/26	6:00 AM	0.016	0.009	0.027	11.0	–	70	50.3	0
4/1/26	12:00 PM	0.018	0.012	0.031	–	–	70	49.3	0.002

Summary Statistics

Date	Time	NO_2	NO	NO_x	$PM_{2.5}$	O_3	Temp	RH	Rain
Minimum		0.002	0.001	0.004	0.1	0.008	32	19.5	0
MinDate		28-Mar	28-Mar	28-Mar	28-Mar	29-Mar	24-Mar	28-Mar	22-Mar
MinTime		06:00	06:00	06:00	06:00	06:00	06:00	06:00	06:00
Maximum		0.037	0.041	0.076	18.8	0.055	78	78	0.302
MaxDate		22-Mar	25-Mar	25-Mar	26-Mar	26-Mar	31-Mar	23-Mar	23-Mar
MaxTime		24:00	12:00	12:00	24:00	06:00	06:00	12:00	06:00
Avg		0.015	0.010	0.027	7.1	0.032	50	46.5	0.494
Num		40	40	40	40	31	41	41	41
Data(%)		97	97	97	97	75	100	100	100
STD		0	0	0	5.1	0	13.1	13.9	–

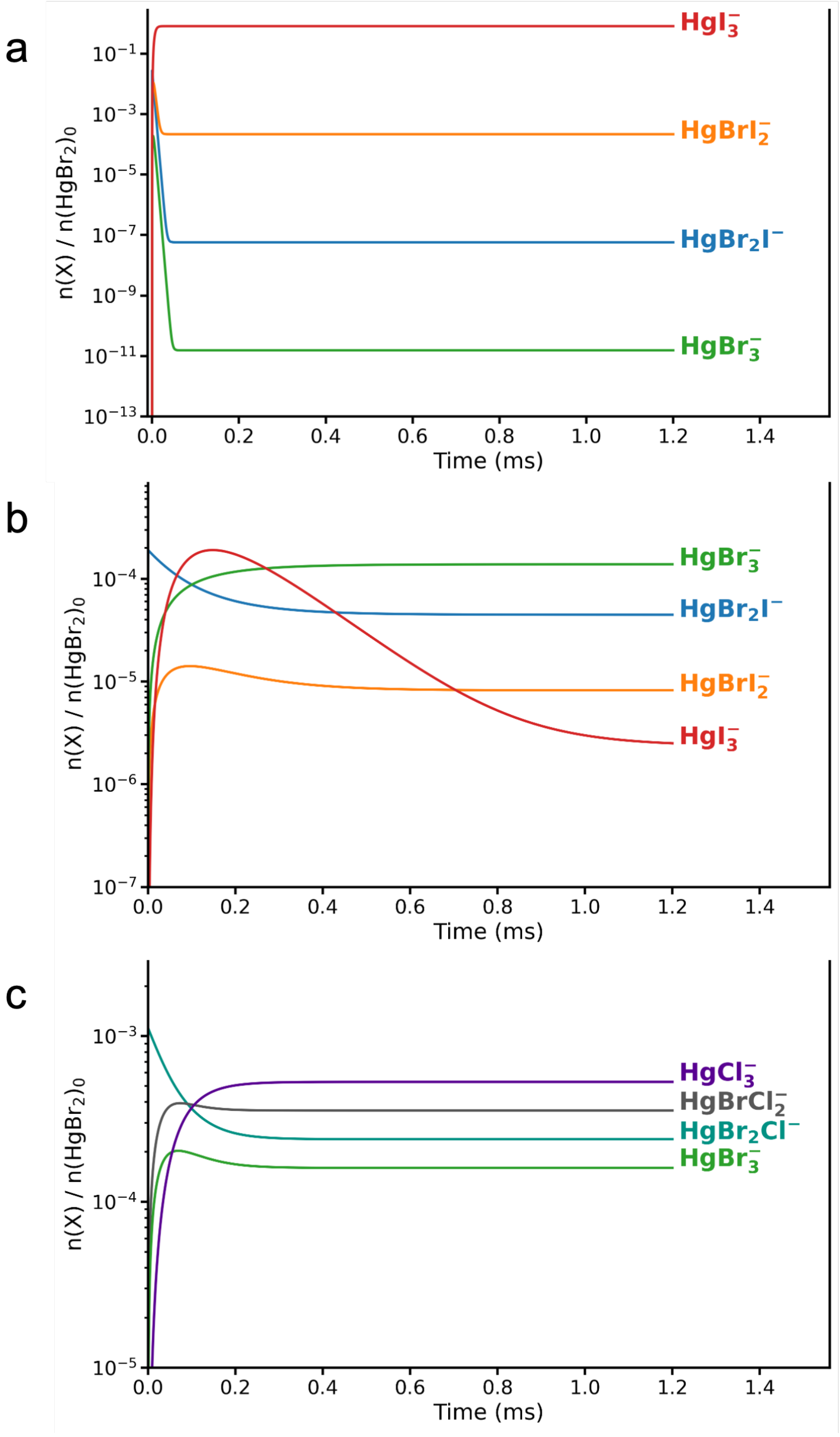

Figure S1: Kinetic-model predictions of halide-mediated speciation pathways for 3.6 ng $HgBr_2$, assuming no droplet evaporation, under three reagent-ion conditions: (a) 26.2 μM NH_4I, (b) 0.16 μM NH_4I, and (c) 59.8 μM NH_4Cl. The primary y-axis represents normalized mole fractions of different species relative to the initial $HgBr_2$ amount, $n(X)/n(HgBr_2)_0$.

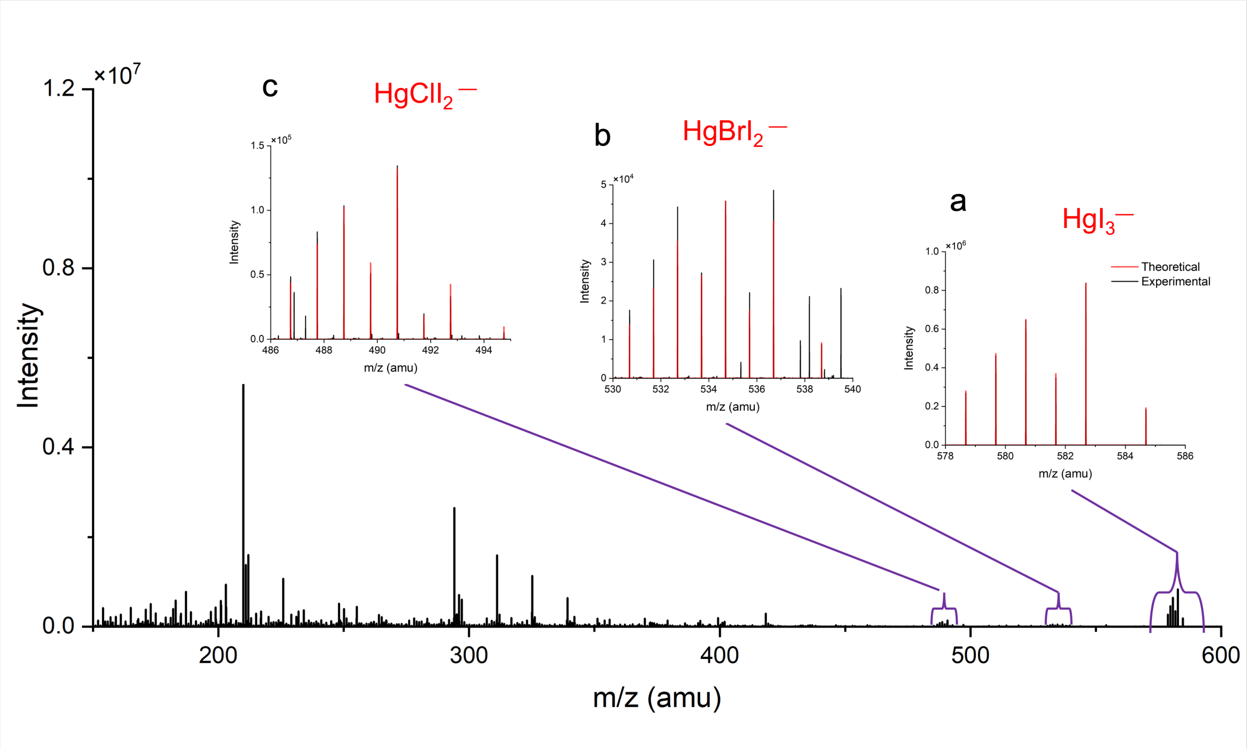

Figure S2: High resolution mass spectra of atmospheric reactive mercury collected on paper over ten days and eluted with 0.15 μM NH_4I: (a) HgI_3^-, (b) $HgBrI_2^-$, and (c) $HgClI_2^-$. The mass spectra are obtained during the second elution step.

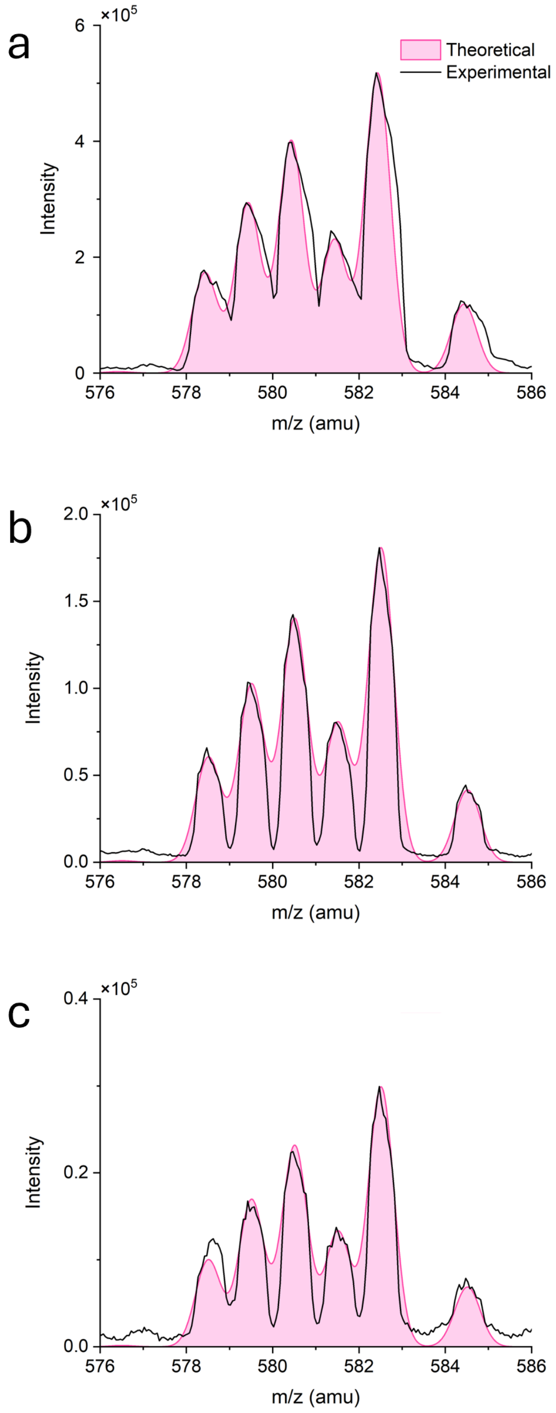

Figure S3: Mass spectra of 14.4 ng $HgBr_2$ elluted with high iodide concentration (26.6 μM), illustrating the influence of matrix effects. Panels (a)–(c) correspond to increasing matrix levels: (a) no matrix, (b) moderate matrix (22.5 μg succinic acid + 18.6 μg $(NH_4)_2SO_4$), and (c) high matrix (225 μg succinic acid + 186 μg $(NH_4)_2SO_4$).

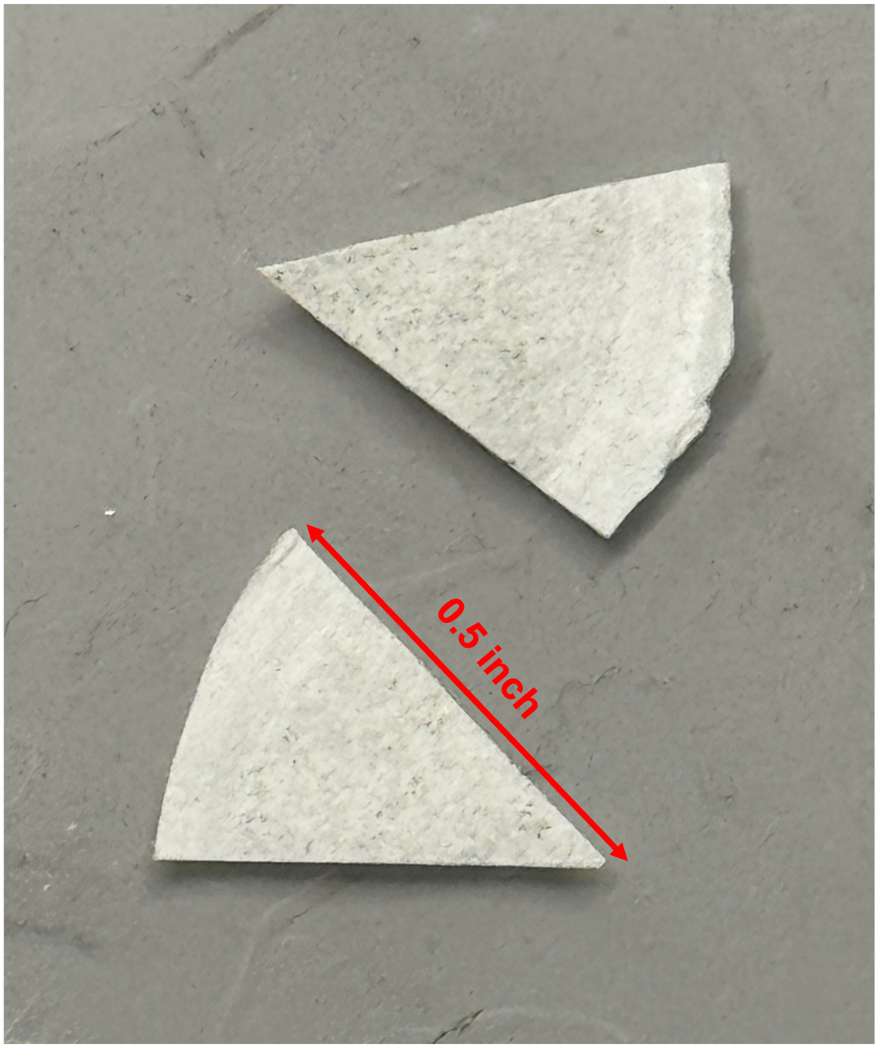

Figure S4: Photograph of the two sectors cut from the sampled paper filter and used for speciation and quantification.

References

(1) Luippold, A.; Gustin, M. S.; Dunham-Cheatham, S. M.; Zhang, L. Improvement of quantification and identification of atmospheric reactive mercury. *Atmospheric Environment* **2020**, *224*, 117307, DOI: https://doi.org/10.1016/j.atmosenv.2020.117307.

(2) Miller, M. B.; Howard, D. A.; Pierce, A. M.; Cook, K. R.; Keywood, M.; Powell, J.; Gustin, M. S.; Edwards, G. C. Atmospheric reactive mercury concentrations in coastal Australia and the Southern Ocean. *Science of The Total Environment* **2021**, *751*, 141681, DOI: https://doi.org/10.1016/j.scitotenv.2020.141681.

(3) Gustin, M. S.; Dunham-Cheatham, S. M.; Allen, N.; Choma, N.; Johnson, W.; Lopez, S.; Russell, A.; Mei, E.; Magand, O.; Dommergue, A.; Elgiar, T. Observations of the chemistry and concentrations of reactive Hg at locations with different ambient air chemistry. *Science of The Total Environment* **2023**, *904*, 166184, DOI: https://doi.org/10.1016/j.scitotenv.2023.166184.

(4) New Jersey Department of Environmental Protection New Jersey Air Quality Index (AQI) and Monitoring Data, https://www.njaqinow.net/default.ltr.aspx.
